# SNP-Based Genotyping Provides Insight Into the West Asian Origin of Russian Local Goats

**DOI:** 10.3389/fgene.2021.708740

**Published:** 2021-07-01

**Authors:** Tatiana E. Deniskova, Arsen V. Dotsev, Marina I. Selionova, Henry Reyer, Johann Sölkner, Margaret S. Fornara, Ali-Magomed M. Aybazov, Klaus Wimmers, Gottfried Brem, Natalia A. Zinovieva

**Affiliations:** ^1^L.K. Ernst Federal Science Center for Animal Husbandry, Podolsk, Russia; ^2^Russian State Agrarian University – Moscow Timiryazev Agricultural Academy, Moscow, Russia; ^3^Institute of Genome Biology, Leibniz Institute for Farm Animal Biology, Dummerstorf, Germany; ^4^Division of Livestock Sciences, University of Natural Resources and Life Sciences, Vienna, Vienna, Austria; ^5^All-Russian Research Institute of Sheep and Goat Breeding – Branch of the Federal State Budgetary Scientific Institution, North Caucasian Agrarian Center, Stavropol, Russia; ^6^Institute of Animal Breeding and Genetics, University of Veterinary Medicine Vienna, Vienna, Austria

**Keywords:** goat, local breeds, single nucleotide polymorphisms, admixture, population structure

## Abstract

Specific local environmental and sociocultural conditions have led to the creation of various goat populations in Russia. National goat diversity includes breeds that have been selected for down and mohair production traits as well as versatile local breeds for which pastoralism is the main management system. Effective preservation and breeding programs for local goat breeds are missing due to the lack of DNA-based data. In this work, we analyzed the genetic diversity and population structure of Russian local goats, including Altai Mountain, Altai White Downy, Dagestan Downy, Dagestan Local, Karachaev, Orenburg, and Soviet Mohair goats, which were genotyped with the Illumina Goat SNP50 BeadChip. In addition, we addressed genetic relationships between local and global goat populations obtained from the AdaptMap project. Russian goats showed a high level of genetic diversity. Although a decrease in historical effective population sizes was revealed, the recent effective population sizes estimated for three generations ago were larger than 100 in all studied populations. The mean runs of homozygosity (ROH) lengths ranged from 79.42 to 183.94 Mb, and the average ROH number varied from 18 to 41. Short ROH segments (<2 Mb) were predominant in all breeds, while the longest ROH class (>16 Mb) was the least frequent. Principal component analysis, Neighbor-Net graph, and Admixture clustering revealed several patterns in Russian local goats. First, a separation of the Karachaev breed from other populations was observed. Moreover, genetic connections between the Orenburg and Altai Mountain breeds were suggested and the Dagestan breeds were found to be admixed with the Soviet Mohair breed. Neighbor-Net analysis and clustering of local and global breeds demonstrated the close genetic relations between Russian local and Turkish breeds that probably resulted from past admixture events through postdomestication routes. Our findings contribute to the understanding of the genetic relationships of goats originating in West Asia and Eurasia and may be used to design breeding programs for local goats to ensure their effective conservation and proper management.

## Introduction

Domestic goats are highly valued for a combination of unique biological, ecological, and economically important characteristics. These easily acclimatizing small ruminants are spread around the world, and Russia is no exception. Archeological data provide indirect evidence that domestic goats were already in the territory of modern Russia 2500 years ago. For example, a goatskin was found in the Pazyryk Tombs (Altai Republic), dating between the fifth and third centuries BC (Alkov, 2007). However, the history of goat breeding in Russia was not well documented until the beginning of the twentieth century.

The popularity of and demand for goats and different goat products varied in the Russian regions. Thus, rearing goats for milk was an unconventional livestock branch and was slowly introduced into agricultural practice in northern Russian provinces during the period of the Russian Empire. Although the benefits and nutritional value of goat milk were actively promoted by local departments of the Russian Society of Goat Breeding, only 19 smallholder farms with 35 goats were organized in 1913 (Beloborodova, 2014).

Down/cashmere and mohair/fibers have always been in steady demand by the population and have been used to knit scarves and shawls and to make high-quality yarn, berets, warm linens, and fine fabrics (Chikalev et al., 2009; Petrov, 2014). “Pautinka,” “spiderweb,” or “wedding ring” shawls, which are famous worldwide for their lightness, softness, warmth, and specialty patterns, are knitted from the down of Orenburg goats, which are raised exclusively in Orenburg Province (Petrov, 2014).

The rapid rise in production resulted from the specific sociocultural and environmental traits of this region and was intricately connected with the history of the city of Orenburg, which was meant to be a key link in trading with Kazakhstan, Central Asia, and India in the eighteenth to nineteenth centuries (Petrov, 2014; Elchina, 2017). The climate in the Orenburg province was harsh, cold, and not favorable to crop production because of frequent locust infestation (Elchina, 2017) and poor soil (Skobeleva and Maksimova, 2008). Therefore, in this time, Cossack families living or having moved to the Orenburg province knitted goat down to various products in order to provide a stable income for their families (Skobeleva and Maksimova, 2008; Elchina, 2017).

In 1851, Orenburg downy shawls received medals at the World Exhibition in London. This contributes to the establishment of the first down-knitting factory in the USSR in 1930 and the first goat breeding state farm (“Guberlinsky”) in 1932 (Skobeleva and Maksimova, 2008; Petrov, 2014). Since 1938, this has been the major breeding farm for Orenburg goats (Petrov, 2014).

Nevertheless, Orenburg goats were poorly acclimatized and produced low quantities of down with short fibers, which was insufficient for large-scale down production (Petrov, 2014). Thus, in the 1930s, to increase mohair and down yield, the aboriginal goats were improved by Angora goats to create the highly productive Soviet Mohair and Altai Mountain breeds (Orekhov, 1989). The new breeds were more adaptable to diverse environmental and feeding conditions and successfully improved local unspecified goat populations in various republics of the USSR (Alkov, 2007). Due to the development of new breeds, down and mohair production became the leading goat breeding branches in the USSR (Orekhov, 1989).

Although the national dairy industry was based mostly on cow milk, the Russian White and Gorky goat breeds were created and became popular among smallholders in the USSR to meet their own consumption needs (Orekhov, 1989). The Russian White breed resulted from long-term folk selection and had a milk yield of 350–550 kg for 200–250 days of lactation. The Gorky breed was developed by improving Russian White goats with the Saanen breed and was characterized by a milk yield of 450–500 kg for 240–300 days of lactation (Novopashina et al., 2019).

No specialized meat breeds were developed in the USSR. The goat-meat industry is based on numerous coarse-wool populations bred by smallholders in all regions (Novopashina et al., 2019). Local goat populations have always been of special importance in the North Caucasian region due to the high proportion of pastures located on extremely steep slopes covered with scarce mountainous vegetation (Mamontova et al., 2011; Zagirov, 2014; Musalaev et al., 2015). Owing to the predominant mountain terrain, pastoralism is a major local livestock management system (Gadzhiev and Bobryshova, 2017). The Karachaev and Dagestan goat populations provide locals with cheap meat, milk, goatskins, and wool (Dolaev, 2009). Local goats have valuable traits such as high adaptability and resilience, hardiness, and resistance to hemosporidiosis and cutaneous gadfly (Musalaev et al., 2015).

The USSR dissolution led to a decrease in the goat population from 3 million head in 1990 (Dolgikh et al., 2012) to fewer than 1.9 million in by the end of 2018 (Dunin et al., 2019b). The patterns of contemporary breed and production types differ from those that were featured before the 1990s. A total of 11 goat breeds have pedigree status and are officially included in the “State Register of Breeding Achievements Approved for Use” in Russia. However, four of the 11 are cosmopolitan breeds, such as Alpine, Saanen, Nubian, and Murciano-Granadina (Ministry of Agriculture of the Russian Federation (MARF)., 2019). Approximately 80% of the goat population is concentrated in three federal districts: the North Caucasus (40%), South (25%), and Siberia (15.5%) (Melnikova, 2016). Based on official records, Saanen (29,770 head) and Soviet Mohair (28,600 head) were the most numerous breeds at the end of 2019 (Dunin et al., 2019a); for more detailed information on the population census of the studied goat breeds, see Supplementary Table 1.

The dairy industry is the most developed goat industry and is based mainly on the use of the highly productive Saanen breed (Novopashina et al., 2019). Under these circumstances, the gene pool of the Russian White and Gorky dairy breeds has been lost as these breeds could not compete with highly productive breeds adapted for rearing in large dairy farms.

Presently, volumes of down and mohair account for 10% of the total amount of processed natural fibers in Russia (Chikalev et al., 2009). The Russian mohair industry is based mostly on the Soviet Mohair breed, which accounts for 40% of the total proportion of all goats on breeding farms and is widespread in the Republic of Tyva and the Republic of Dagestan (Novopashina et al., 2019). The closure of the last down-knitting factory in 1995 led to dramatic consequences in the down-knitting trade and to decrease of Orenburg goat population (Skobeleva and Maksimova, 2008). Recently, to support the down and mohair industries, several strategies have been initiated, including rebranding Orenburg shawls and presenting designer outfits made from down and mohair at fashion shows (Yanshina et al., 2018). However, these attempts have not been sufficient to facilitate increased numbers of Orenburg and Altai Mountain, which have been neglected for the last two decades and are probably endangered (Novopashina et al., 2019).

In addition, no data on the origin, developmental history, and admixture levels of local pastoral breeds are available, which inhibits the development of effective programs for their conservation and sufficient management.

Genetic investigations of goats reared in Russia are limited to a few studies. The influence of CAST and BLG genes on economically important traits has been studied in goat populations bred in the Altai Republic (Goncharenko et al., 2018). The genetic diversity of several Russian breeds has been determined using microsatellites (Petrov et al., 2018; Kharzinova et al., 2019) and mtDNA D-loop polymorphisms (Deniskova et al., 2020a). In addition, based on SNP data, the population structure of Saanen goats of Russia has been evaluated (Deniskova et al., 2020b) and some genetic parameters in local goat populations were calculated (Deniskova et al., 2020c). However, the lack of data on the current state of the gene pool of Russian goats based on the implementation of genomic technologies is the main obstacle to developing efficient breeding and conservation programs for local breeds.

The introduction of the 50 K array (International Goat Genome Consortium (IGGC)., 2013; Tosser-Klopp et al., 2014) has promoted a rise in genomic studies of goats belonging to local (Nicoloso et al., 2015; Manunza et al., 2016; Talenti et al., 2017) and cosmopolitan breeds (Visser et al., 2016; Brito et al., 2017). Along with classical diversity indicators, estimation of effective population size and assessment of patterns of the runs of homozygosity (ROH) distribution are essential to address the demographic history and to evaluate the extent of inbreeding at genome level in local goats (Onzima et al., 2018; Islam et al., 2019; Michailidou et al., 2019).

On the basis of SNP profiles from the AdaptMap project (Stella et al., 2018), a significant scientific breakthrough has been made in understanding the migration events of goat breeds worldwide (Colli et al., 2018a) and identifying the regions that underlie artificial and environmental selection (Bertolini et al., 2018b). Due to the availability and standardized genotyping protocol, the AdaptMap dataset enables us to address origin and to establish genetic links between local and global goat populations (Michailidou et al., 2019).

In this regard, the aims of this research work were to (i) assess genetic diversity and population structure, (ii) study national genetic variation, and (iii) determine the genetic position of Russian local goats among goat populations around the world obtained from the AdaptMap project.

## Materials and Methods

### Ethics Approval

The reported study was performed in accordance with the ethical guidelines of the L.K. Ernst Federal Research Center for Animal Husbandry. The protocol was approved by the Commission on the Ethics of Animal Experiments of the L.K. Ernst Federal Research Center for Animal Husbandry. The animal tissue samples were collected by trained personnel under strict veterinary rules in accordance with the rules for conducting laboratory research (tests) in the implementation of the veterinary control (supervision) approved by Council Decision Eurasian Economic Commission no 80 (November 10, 2017).

### Sample Collection

For this study, tissue samples from Altai Mountain (*n* = 33), Altai White Downy (*n* = 20), Dagestan Downy (*n* = 34), Dagestan Local (*n* = 20), Karachaev (*n* = 36), Orenburg (*n* = 32), Saanen (*n* = 33), and Soviet Mohair (*n* = 29) goat breeds were collected from 2017 to 2019.

The sampling covered the most important goat breeding regions in Russia. A brief description of specific sampling points with geographical coordinates is given in Supplementary Table 2. A description of the sample collection for this study is presented in Table 1.

**TABLE 1:** Sample collection of goats for this study.

**Breed**	**Breed code**	**Period of sampling**	***n***	***n* of flocks**	***n* per flock**	**Farm type**	**Sampling region**
Altai mountain	ALTM	2018–2019	33	2	8	Breeding farm	Altai Republic
					25	Breeding farm	Altai Republic
Altai white downy	ALTW	2019	20	1	20	Breeding farm	Altai Republic
Dagestan downy	DAGD	2018–2019	34	1	34	Smallholders	Republic of Dagestan
Dagestan local	DAGL	2018–2019	20	2	10	Smallholders	Republic of Dagestan
					10	Smallholders	Republic of Dagestan
Karachaev	KARA	2018–2019	36	3	12	Smallholders	Karachay-Cherkessia Republic
					10	Smallholders	Karachay-Cherkessia Republic
					14	Smallholders	Karachay-Cherkessia Republic
Orenburg	OREN	2017–2019	32	2	19	Breeding farm	Orenburg region
					13	Breeding farm	Orenburg region
Saanen	SAAN	2018–2019	33	2	18	Nucleus farm	Leningrad region
					15	Breeding farm	Stavropol region
Soviet mohair	SOVM	2018–2019	29	2	8	Breeding farm	Republic of Tyva
					21	Breeding farm	Republic of Tyva

*n, number of individuals; n of flocks, number of flocks sampled; n per flock, number of individuals sampled per flock.*

Sampling was performed on various types of farms, including breeding, nucleus, and smallholder farms. Except for the Altai White Downy and Dagestan Downy breeds, goats from each breed were sampled from two or more flocks. In our study, the samples of the Altai White Downy and Dagestan Downy breeds were collected from one flock for each breed. The Altai White Downy is new breed which was officially established only in 2016 and is bred currently only at a single farm. There is an opposite situation with the Dagestan Downy breed which is endangered. The small number of purebred goats of this breed is kept only in a single farm.

The choice of goats for this study was based on specific criteria. Only typical individuals that met the officially established breed standards were selected. The average age of the goats per flock varied from 1.8 to 2.1 years. Body weight deviation and withers height deviation among selected animals within each flock did not exceed 10–12 and 5%, respectively.

**FIGURE 1:**
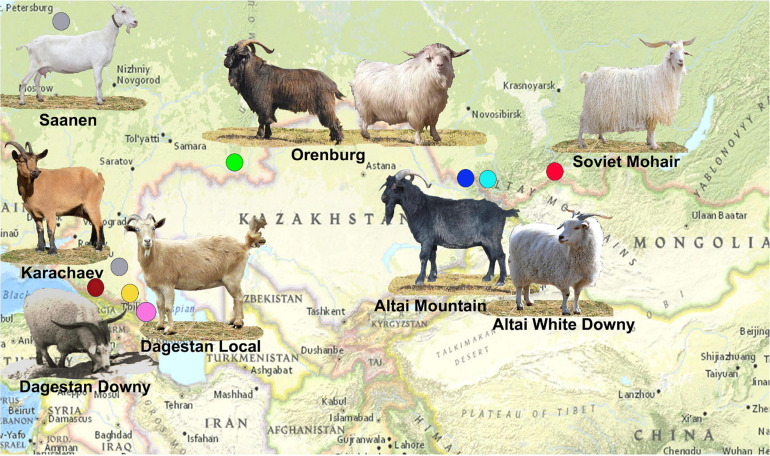
Map illustrating the area of sampling for Russian goat populations. The sampling locations are shown as circles colored blue for Altai Mountain, cyan for Altai White Mountain, yellow for Dagestan Downy, pink for Dagestan Local, brown for Karachaev, green for Orenburg, gray for Saanen, and red for Soviet Mohair. The Orenburg breed is shown in two coat color variations. The photography of the Dagestan Downy goat was provided by Dr. Musalaev Khanmagomed and Dr. Rashid Abdullabekov (Federal Agrarian Scientific Center of the Republic of Dagestan). The photographs of the black Orenburg, Altai Mountain, and Soviet Mohair goats were taken by Irina Pridanova and obtained from Dunin and Dankvert (2013).

A map illustrating the area of sampling for Russian goat populations is shown in Figure 1.

### Genotyping and Quality Control

Genomic DNA was extracted with standard protocols using DNA kits produced by Synthol Company (Moscow, Russia). All goats were genotyped with the Illumina Goat SNP50 BeadChip (International Goat Genome Consortium (IGGC)., 2013; Tosser-Klopp et al., 2014).

Quality control was performed by setting a cutoff of 0.5 for the GenCall and GenTrain scores (Fan et al., 2003). We excluded samples for which less than 90% were genotyped (–mind 0.1) from the analysis. Furthermore, we discarded SNPs for which less than 90% of the samples were called in (–geno 0.1), those with a minor allele frequency (MAF) lower than 5% (–maf 0.05), and those located on sex chromosomes and with unknown positions. For ROH-based analyses, the MAF filter was not applied.

A unified additive relationship matrix according to Yang et al. (2010) was estimated using the R package “snpReady” (Granato and Fritsche-Neto, 2018) to avoid possible biases related to family structures. Highly related animals (relationship >0.35) were excluded from further analysis. A cutoff of 0.5 was set for the Saanen breed because samples were collected in farms with a significant number of close relatives.

### Construction of the Working Datasets

In our study, we constructed three working datasets. The first one included seven Russian local breeds and the Saanen breed, which are reared in Russia, to study variation within national goat populations.

To infer the genetic relationships and to address admixture patterns of Russian goats, we combined the SNP profiles from the first working dataset with those representing goat populations from different parts of the world generated in the framework of the AdaptMap Project (Stella et al., 2018). The data were downloaded from Data Dryad (Colli et al., 2018b).

The second working dataset was used to construct a Neighbor-Net graph and, in addition to Russian goat populations, contained 105 breeds from the AdaptMap dataset, including 33 populations from Europe, 41 from Africa, 19 from West Asia, two from North America, three from South America, and two from Oceania as well as five cosmopolitan breeds (Angora, Boer, Malya, Nubian, and Saanen).

To more precisely study the genomic composition of Russian local breeds, we used breeds that were included in the same or neighboring clusters with Russian goats in the Neighbor-Net graph as well as cosmopolitan breeds. Therefore, the third working dataset comprised 38 breeds, including eight populations from Russia, one from Europe, four from Africa, 18 from West Asia, and two from Oceania as well as five cosmopolitan breeds from the AdaptMap dataset.

Details on the geographical distribution and full names of breeds, which were used to perform neighbor network analysis and clustering with Admixture software, are presented in Supplementary Table 3.

### Genetic Diversity Estimation

In the R package “diveRsity” (Keenan et al., 2013), the observed heterozygosity (Ho), unbiased expected heterozygosity (H_*E*_), and inbreeding coefficient (Fis) with 95% confidence interval (CI 95%) were calculated to evaluate within-population genetic diversity.

### Runs of Homozygosity (ROH) and Genomic Inbreeding (F_*ROH*_)

A consecutive runs method (Marras et al., 2014) implemented in the R package “detectRUNS” (Biscarini et al., 2018) was used to estimate ROH. One SNP with a missing genotype and up to one possible heterozygous genotype was allowed in the run. The minimum ROH length was 1000 kb. To decrease false positive results, the minimum number of SNPs (l) was calculated, as was initially proposed by Lencz et al. (2007) and followed by Purfield et al. (2012) in a study on cattle breeds:

(1) l=[log(α/n×sn)ie]/[log(1-het)e],

where n_*s*_ = the number of genotyped SNPs per individual; n_*i*_ = the number of genotyped individuals; α = the percentage of false positive ROH (set to 0.05 in our study); and het = the mean heterozygosity across all SNPs. The calculated *l* was equal to 17.

We estimated ROH for each goat and then categorized ROH in the following length classes: 1–2, 2–4, 4–8, 8–16, and >16 Mb.

We computed the total number of identified ROH for each length category in each individual of each breed. The mean sum of ROH was calculated by adding the length of all ROH for each animal in the goat populations and then averaging the results per breed population.

The genomic inbreeding coefficient based on ROH (F_*ROH*_) was computed as the sum of the length of all ROH per goat as a proportion of the total autosomal SNP coverage.

### Effective Population Sizes

We estimated trends of effective population size (*Ne*) from linkage disequilibrium (LD) in the *SNeP* software (Barbato et al., 2015). Default parameters were applied, except for the sample size correction, occurrence of mutation (α = 2.2; Corbin et al., 2012), and recombination rate between a pair of genetic markers according to Sved and Feldman (1973).

The most recent estimate of *Ne* was taken three generations ago (*Ne*_3_) to evaluate the modern status of the breeds. The values of *Ne* for five (*Ne*_5_), 10 (*Ne*_10_), and 13 (*Ne*_13_) generations ago were estimated to make our data comparable with previous studies on different goat breeds (Brito et al., 2015; Visser et al., 2016; Michailidou et al., 2019; Monau et al., 2020).

### Genetic Relationship and Population Structure

Pairwise genetic differentiation (*F*_*ST*_) (Weir and Cockerham, 1984) was calculated using the R package StAMPP (Pembleton et al., 2013). Reynolds distances (Reynolds et al., 1983) were calculated in the R package adegenet (Jombart and Ahmed, 2011).

Principal component analysis (PCA) was performed in PLINK v1.9 (Chang et al., 2015) and visualized with the R package “ggplot2” (Wickham, 2009).

The Neighbor-Net graphs, based either on the matrix of pairwise *F*_*ST*_ values for the first working dataset or on the matrix of pairwise Reynolds distances for the second working dataset, were constructed in SplitsTree 4.14.5 software (Huson and Bryant, 2006).

Genomic clustering was performed separately for the first and third working datasets using Admixture v1.3 software (Alexander et al., 2009) and plotted with the R packages “pophelper” (Francis, 2017) and “BITE” (Milanesi et al., 2017), respectively. The choice of K was based on the lowest cross-validation error compared to other *K* values as implemented in a standard Admixture cross-validation procedure (Alexander et al., 2009).

The map illustrating the area of sampling for each Russian goat breed was obtained from the NatGeo Mapmaker Interactive Database (NMID).

## Results

### Genetic Diversity and Effective Population Sizes in Russian Goat Populations

Genetic diversity indicators and effective population sizes are summarized in Table 2.

**TABLE 2:** Diversity parameters and effective population sizes in Russian goat populations.

**Breed**	**Code**	***n***	**H_*o*_**	**H_*E*_**	**Fis [CI 95%]**	***Ne*_3_**	***Ne*_5_**	***Ne*_10_**	***Ne*_50_**
Altai mountain	ALTM	33	0.398	0.405	0.019 [0.017; 0.021]	241	291	398	1007
Altai white downy	ALTW	20	0.402	0.400	−0.006 [−0.008; −0.004]	133	163	233	683
Dagestan downy	DAGD	34	0.402	0.413	0.025 [0.023; 0.027]	342	445	657	1825
Dagestan local	DAGL	20	0.413	0.426	0.03 [0.028; 0.032]	241	381	657	2528
Karachaev	KARA	36	0.386	0.389	0.006 [0.004; 0.008]	190	184	205	519
Orenburg	OREN	32	0.403	0.403	0.001 [−0.001; 0.003]	414	511	600	1226
Saanen	SAAN	33	0.413	0.421	0.017 [0.015; 0.019]	154	199	277	849
Soviet mohair	SOVM	29	0.400	0.400	0 [−0.002; 0.002]	270	366	510	850

*n, number of individuals; H_*o*_, observed heterozygosity; H_*E*_, unbiased expected heterozygosity; Fis, inbreeding coefficient with 95% confidence interval [CI 95%]; *Ne_3,_ Ne_5_ Ne_10_*, and *Ne*_50_, effective population sizes three, five, 10, and 50 generations ago, respectively. Standard errors (se) were ±0.001 for H_*o*_ and H_*E*_.*

The average values of observed and expected heterozygosity were 0.402 and 0.407, respectively, varying from minima in the Karachaev (H_*o*_ = 0.386; H_*E*_ = 0.389) to maxima in the Dagestan Local (H_*o*_ = 0.434; H_*E*_ = 0.417) and Saanen breeds (H_*o*_ = 0.413; H_*E*_ = 0.421).

Estimates of the inbreeding coefficient were significant at the 95% confidence interval and moderately positive in the Altai Mountain, Dagestan Downy, Dagestan Local, Karachaev, and Saanen breeds. For the other breeds, estimates of the inbreeding coefficient were not significant.

Values of the recent effective population sizes estimated for three and five generations ago were the highest in the Dagestan Downy and Orenburg breeds. The Altai White Downy and Saanen breeds displayed the lowest values. A tendency toward a smooth decrease in effective population sizes was apparent in all Russian goat groups within 60 generations (Supplementary Figure 1). However, the recent effective population size estimated for three generations ago for the Karachaev breed is higher than that calculated for five generations ago. The values of historical effective population sizes estimated for 50 generations ago varied from 519 in the Karachaev breed to 2528 in the Dagestan Local breed.

More specific patterns were obtained by expanding the number of generations up to 520 (Supplementary Figure 2). Thus, the changes in *Ne* sizes in the Dagestan Local breed were more dramatic than the *Ne* trends detected in other goat breeds. In this breed, there were peaks at 506 (*Ne* = 12603) and 430 (*Ne* = 11048) generations ago, with an intermediate decline in *Ne* size 477 (*Ne* = 9583) generations ago. In addition, a peak was found in the Altai Mountain breed 506 (*Ne_506_* = 5948 versus *Ne_520_* = 5757 and *Ne_477_* = 4959) generations ago.

### The Pattern of Distribution of ROH in Russian Goat Populations

**FIGURE 2:**
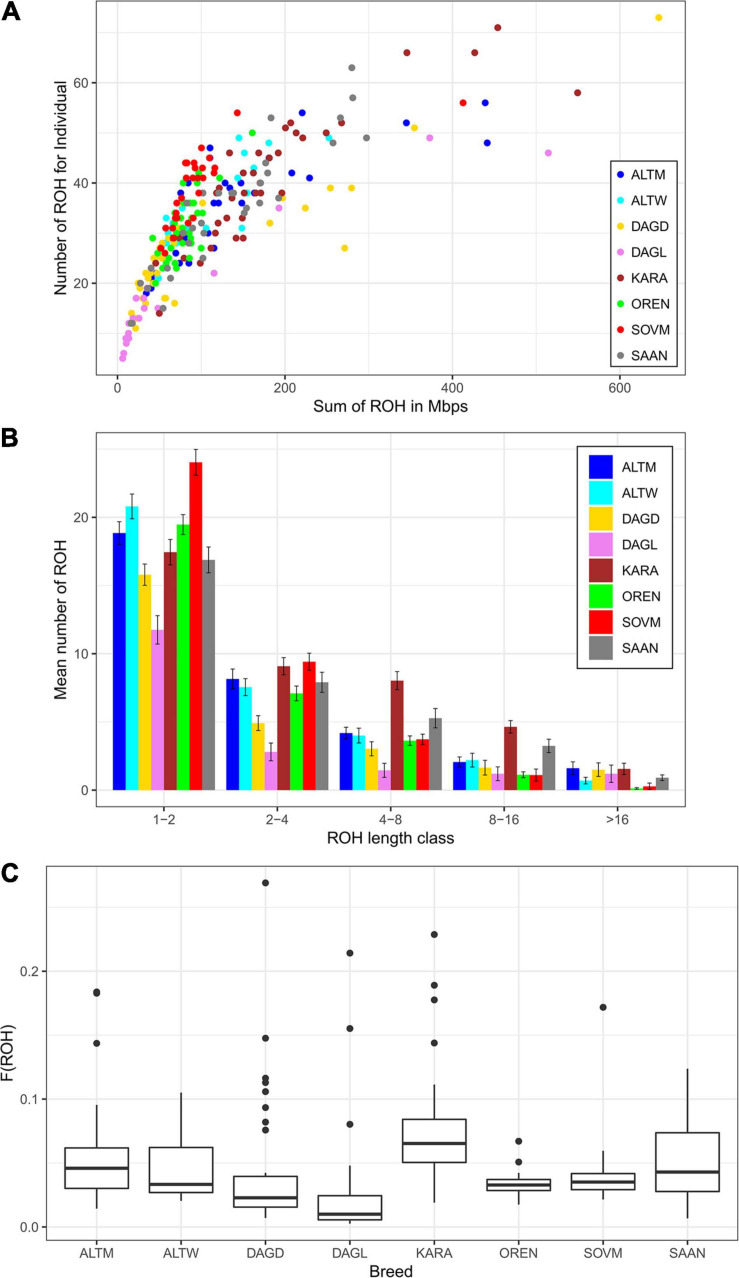
Patterns of runs of homozygosity (ROH) in Russian goat populations: genomic coverage in ROH (*X*-axis) and ROH number per individual (*Y*-axis) **(A)**, ROH distribution in length classes **(B)**, and variations of the runs of homozygosity inbreeding coefficient (F_*ROH*_) within each goat group **(C)**. For a description of the goat breeds, see Table 1.

The ROH segments were identified in all breeds, with mean lengths ranging from 79.42 Mb in the Dagestan Local to 183.94 Mb in the Karachaev breed and with average ROH numbers varying from 18.4 to 40.75 in Dagestan Local and Karachaev breeds, respectively. The maximum individual ROH length and ROH number were found in the Dagestan Downy breed (646.47 Mb and 73, respectively), while the Dagestan Local breed displayed the lowest values (6.17 Mb and 5, respectively; Table 3). Nevertheless, most goats demonstrated similar patterns of individual homozygosity (number of ROH segments of 60 and genome coverage of 200–250 Mb; Figure 2A).

**TABLE 3:** Mean ROH length and mean ROH number in Russian goat populations.

**Breed**	**Code**	***n***	**ROH length**			**ROH number**		
			**Mean**	**Min**	**Max**	**Mean**	**Min**	**Max**
Altai mountain	ALTM	33	134.2117.9	34.4	441.56	34.851.81	18	56
Altai white downy	ALTW	20	107.9312.46	49.01	252.47	35.251.81	21	49
Dagestan downy	DAGD	34	107.6422,31	16.8	646.47	26.882.07	11	73
Dagestan local	DAGL	20	79.4230.1	6.17	514.53	18.42.88	5	49
Karachaev	KARA	36	183.9418.06	45.65	549.49	40.752.12	14	71
Orenburg	OREN	32	80.384.07	42	161.01	31.441.18	20	50
Saanen	SAAN	33	129.7214.03	16.08	297.39	34.212.21	12	63
Soviet Mohair	SOVM	29	96.9611.9	51.61	412.71	38.551.4	26	56

*n, number of individuals; Min, minimum values of estimations observed in individual animals within breeds; Max, maximum values of estimations observed in individual animals within breeds.*

Considering the length category distribution, the shortest ROH segments (1–2 Mb) were predominant in all studied breeds, with variation from 42.81% in the Karachaev breed to 63.86% in the Dagestan Local breed (Figure 2B). The distribution of the 2–4 and 4–8 Mb ROH length classes varied from 15.22% (Dagestan Local) to 24.42% (Soviet Mohair) and from 7.88% (Dagestan Local) to 19.70% (Karachaev), respectively. The frequencies of the long ROH segments (8–16 Mb) ranged from 2.86% in the Soviet Mohair breed to 11.38% in the Karachaev breed. The frequencies of the longest ROH segments (>16 Mb) varied from the rarest in the Orenburg and Soviet Mohair (0.40–0.72%) breeds to low in the Altai White Downy, Saanen, and Karachaev (1.99–3.82%) to moderate in the Altai Mountain, Dagestan Downy, and Dagestan Local (4.61–6.52%) breeds.

All goat breeds display low mean values of F_*ROH*_, varying from 0.033 in the Dagestan Local and Orenburg breeds to 0.077 in the Karachaev breed (Figure 2C). The highest individual levels of F_*ROH*_ are found in the Dagestan Downy (*F*_*ROH*_ = 0.27), Karachaev (*F*_*ROH*_ = 0.23), and Dagestan Local breeds (*F*_*ROH*_ = 0.21). Some goats from the Altai Mountain, Dagestan Local, Dagestan Downy, and Saanen breeds had F_*ROH*_ estimates lower than 0.01.

### Genetic Relations and Population Structure Among Russian Goats

**FIGURE 3:**
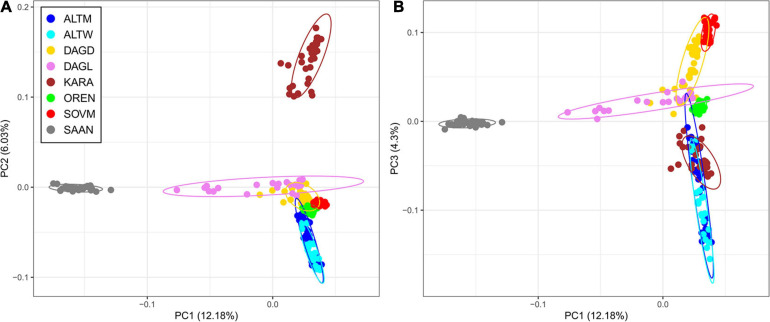
Principal component analysis for Russian goat populations. The analysis was performed for the first two principal components (PC1 and PC2) **(A)** and for the first and third principal components (PC1 and PC3) **(B)**. For a description of the goat breeds, see Table 1.

Principal component analysis showed that the Saanen breed and several goats belonging to the Dagestan Local and Dagestan Downy breeds are separated from Russian local breeds by PC1, accounting for 12.18% of genetic variability (Figure 3A). PC2, accounting for 6.03% of the genetic variability, divides the Karachaev breed from the other studied breeds. PC3 demonstrates a clustering of the Karachaev breed with other Russian local goats and shows a separated distribution of the Orenburg and Soviet Mohair breeds (Figure 3B).

A Neighbor-Net graph based on pairwise *F*_*ST*_ distances between Russian goat populations demonstrates that branches of two breeds are not directly connect to the net (Supplementary Figure 3). Thus, the Saanen breed is linked to the net of Russian local goats through the Dagestan Local breed, while the short branch of the Altai White Downy breed is attached to the net via the Altai Mountain breed. The Orenburg and Altai Mountain, Dagestan Downy, and Soviet Mohair are closely related, and as well as Dagestan Local and Karachaev breeds. The Dagestan Downy breed is located on the net edge.

The calculated *F*_*ST*_ values between the Saanen and Russian local breeds were moderate and varied from 0.06 (Dagestan Local) to 0.11 (Karachaev). Among Russian local goats, moderate differentiation was recorded between the Karachaev breed and the following breeds: Altai White Downy (*F*_*ST*_ = 0.063), Soviet Mohair (*F*_*ST*_ = 0.057), Orenburg (*F*_*ST*_ = 0.056), and Altai Mountain (*F*_*ST*_ = 0.055). For the other pairs of breeds, the *F*_*ST*_ distances corresponded to low differentiation. The minimal *F*_*ST*_ values were found between the Altai White Downy and Altai Mountain (*F*_*ST*_ = 0.003), Dagestan Downy and Dagestan Local (*F*_*ST*_ = 0.011), and Soviet Mohair and Dagestan Downy (*F*_*ST*_ = 0.017) breeds.

**FIGURE 4:**
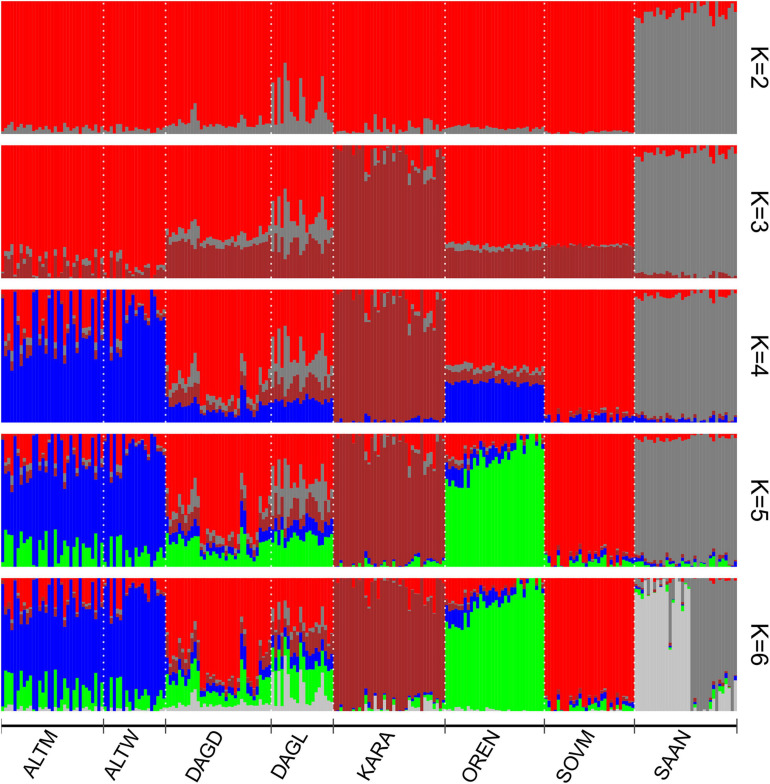
Population structure of Russian goat populations. For a description of the goat breeds, see Table 1.

Clustering with Admixture software shows a clear differentiation of the Saanen breed from Russian local goats beginning at *K* = 2 (Figure 4). The Karachaev breed is the first to be subdivided within Russian local populations (*K* = 3). A *K*-value equal to 6 had the lowest cross-validation error (Supplementary Figure 4). At *K* = 6, the Orenburg, Soviet Mohair, and Karachaev breeds have their own clusters. The Altai Mountain and Altai White Downy breeds reveal a shared genomic background with admixture traces from the Soviet Mohair and Orenburg breeds. The Saanen breed was divided into two groups. Both Dagestan breeds are of admixed origin. Soviet Mohair genetic influence is predominant in the Dagestan Downy breed. The Dagestan Local breed has a stronger admixture from the Saanen breed and demonstrates the presence of genomic components that are predominant in the Karachaev breed.

### Assessing the Ancestry, Population Structure, and Phylogenetic Links of Russian Goats in the Context of Goat Breeds Included in the AdaptMap Project

**FIGURE 5:**
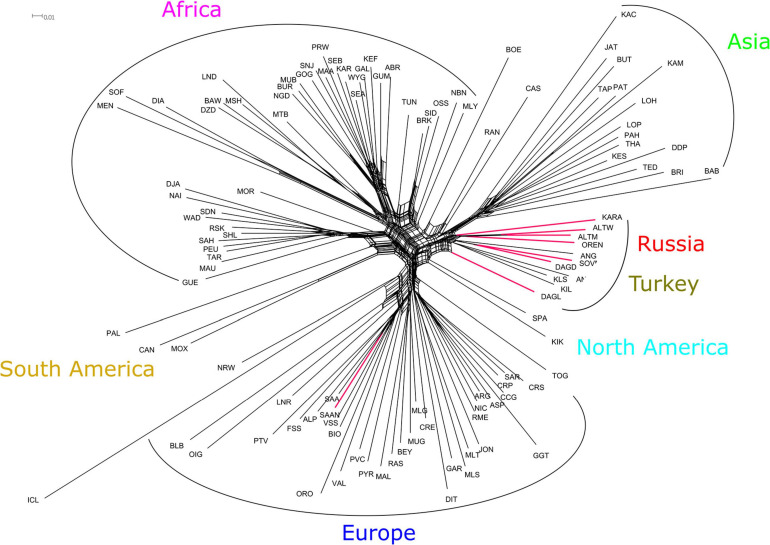
Neighbor-Net graph based on Reynolds distances for Russian and worldwide goat populations from AdaptMap. Branches for Russian local goats are colored red. For a description of the goat breeds, see Table 1.

The Neighbor-Net graph based on Reynolds distances between Russian and worldwide goat populations showed that Russian local and Turkish breeds form a genetic cluster (Figure 5). The Dagestan Local breed had an independent branch and was the most distant within the relevant genetic cluster. The Angora, Soviet Mohair, and Dagestan Downy breeds clustered together. The second cluster included the remaining four Russian local breeds (Orenburg, Altai Mountain, Altai White Downy, and Karachaev). The third group comprised Turkish breeds (Ankara, Kil, and Kilis). The Saanen population collected for this study in Russian regions was clustered with a Saanen (Switzerland) population from the AdaptMap project.

**FIGURE 6:**
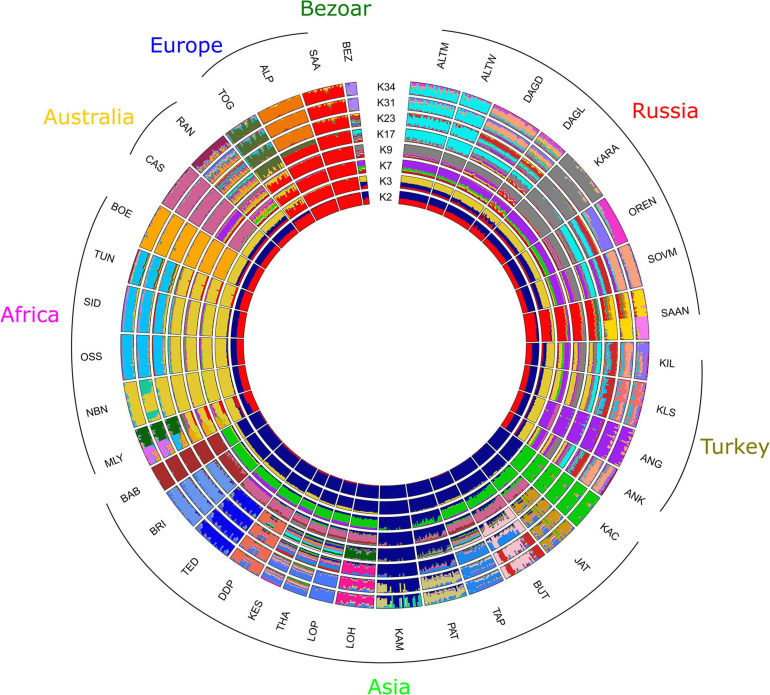
Clustering of Russian goat populations in the context of the dataset of worldwide goat populations from AdaptMap. For a description of the goat breeds, see Table 1.

Admixture analysis performed for Russian and worldwide goat populations from the AdaptMap set showed that at *K* = 2, local Russian breeds demonstrated admixed West Asian and European origin, while the Saanen breeds showed European ancestry (Figure 6). At *K* = 3, Russian local goats showed a genomic composition similar to that of the Turkish and Cashmere breeds as well as the Bezoar. The definite similarity with the abovementioned breeds was still present at *K* = 7. However, Russian local goats showed a larger influence of a genetic background that was predominant in West Asian breeds (green color). At *K* = 9, a new specific genomic component (gray color) was exhibited in all Russian local goats and prevailed in the Karachaev breed. At *K* = 17, the Altai Mountain and Altai White Downy breeds formed a cluster, with genomic elements (cyan color) that were also found in the Orenburg and Soviet Mohair breeds as well as in several Turkish breeds. At *K* = 23, the Saanen breed from this study was divided into two groups. At *K* = 31, the Orenburg breed showed a specific genetic background (lavender color). At *K* = 34, with the lowest cross-validation error (Supplementary Figure 5), the Orenburg (pink color) and Karachaev (gray color) breeds have their own clusters, while the Altai Mountain and Altai White Downy breeds form a shared group (cyan color). The genomic composition that is predominant in the Soviet Mohair breed (light salmon color) is also found in the Dagestan Downy, Dagestan Local, and several Turkish breeds.

## Discussion

### Genetic Diversity, Effective Population Sizes, and ROH Distribution

Compared to other livestock species, the genetic resources of local goats are among the most underestimated and require proper maintenance, rational utilization, and preservation for future generations (Novopashina et al., 2019). Therefore, we analyzed the genetic diversity indicators, as well as patterns of homozygosity, and estimated effective population sizes to evaluate the state of the genetic resources of local goats in Russia.

Genetic diversity indicators in Russian goat populations are compatible with those obtained for cosmopolitan breeds, including the Angora (Visser et al., 2016), Boer, and Saanen (Colli et al., 2018a) breeds, and for native breeds including Italian (Nicoloso et al., 2015), Greek (Michailidou et al., 2019), Ugandan (Onzima et al., 2018), and Sudanese goat populations (Rahmatalla et al., 2017). In addition, H_*o*_ and H_*E*_ estimates in our research exceed the average values calculated for Europe and West Asia (Colli et al., 2018a). Colli et al. (2018a) suggested that pastoralism has frequently led to increased levels of observed heterozygosity in goat breeds. In our study, goat groups for which transhumance is the predominant management system (two Dagestan and Karachaev populations) as well as breeds selected for specific production traits, including Altai Mountain, Orenburg, Soviet Mohair, and Saanen, display high heterozygosity values.

The effective population sizes of Russian local goat breeds have decreased through generations, which corresponds to the global trend for goat breeds in different parts of the world (Brito et al., 2015; Visser et al., 2016; Colli et al., 2018a; Islam et al., 2019) and for other livestock species (Makina et al., 2015; Kominakis et al., 2017). A more dramatic decline in *Ne* was recorded in the last 100 generations in all Russian breeds, as was previously reported in other goat breeds (Brito et al., 2015; Visser et al., 2016; Colli et al., 2018a; Islam et al., 2019). Accordingly, *Ne*_3_ decreased by five to nine times in the Orenburg and Dagestan Downy breeds and by 18–19 times in the Dagestan Local breed in comparison with *Ne*_103_ values. However, the analysis of recent effective population sizes measured at five, 10, and 13 generations ago in native and cosmopolitan world goat breeds demonstrates that the values estimated in Russian populations are not considered low. Thus, effective population sizes at 13 generations ago vary from 87 for Boer to 266 for Tswana goats (Monau et al., 2020), from 52 for Booted goats to 157 for Chamois Colored goats (Burren et al., 2016), and from 96 for Eghoria goats to 127 for Skopelos goats (Michailidou et al., 2019) (*Ne*_13_ from 223 to 796 in our study). The Angora subpopulations are characterized by *Ne*_10_ ranging from 57 to 93 (Visser et al., 2016), while the lowest *Ne*_10_ is equal to 205 in the present work. Effective population sizes at five generations ago in Russian local goats are higher than those obtained in cosmopolitan breeds such as Saanen, Cashmere, LaMancha, Toggenburg, and Nubian and comparable with the *Ne*_5_ value of the Alpine breed (Brito et al., 2015). Effective population sizes at five generations ago in the Saanen breed in our study exceeded the values estimated in the Saanen breed reared in Canada (Brito et al., 2015): 199 and 113, respectively.

Increased effective population sizes of Russian local goat breeds in comparison with cosmopolitan breeds might be a consequence of the absence of artificial insemination in the breed development process and the application of less intensive managing systems. This assumption is partially consistent with the results obtained by Colli et al. (2018a), which demonstrated that *Ne* might be higher in extensively managed local breeds than in more intensively managed goat populations. Thus, current effective population sizes in Russian local breeds exceed the threshold of *Ne* = 100 (Meuwissen, 2009), which ensures the maintenance of these populations.

The analysis of patterns of homozygosity provides insight into demographic history. In addition, evaluation of ROH length allows us to assess the presence of long-term inbreeding in livestock populations (Purfield et al., 2012; Curik et al., 2014). The mean ROH number and mean ROH coverage in Russian local goat breeds (18–41 ROH and 79.42–183.94 Mb) are lower than those in both Turkish breeds (60 ROH and 210.64 Mb) and Central Asian breeds (90 ROH and 260.64 Mb) (Bertolini et al., 2018a).

Usually, ROH number and ROH coverage are larger in highly selected breeds in comparison with native breeds (Bertolini et al., 2018a). In our study, Dagestan Local breed is characterized by lower ROH number compared to breeds selected for specific trait such as Orenburg, Altai Mountain, Soviet Mohair, and Saanen. Nonetheless, the Karachaev population demonstrates the highest ROH coverage among local goats. Bertolini et al. (2018a) also observed a pattern of increased homozygosity that has occurred in local breeds due to small population size and geographic isolation.

Russian goat populations have the largest proportion of short ROH segments (<4 Mb) among other length categories. A high number of short ROH segments frequently result from ancestral family relatedness (Kirin et al., 2010; Bertolini et al., 2018a), as was reported in goats at the worldwide scale (Bertolini et al., 2018a) and at the local scale (Burren et al., 2016; Onzima et al., 2018; Islam et al., 2019; Michailidou et al., 2019). The presence of long ROH segments might be a consequence of artificial selection as well as demographic declines or recent inbreeding (Purfield et al., 2012; Kim et al., 2013; Bertolini et al., 2018a). In our study, the Dagestan Downy, Dagestan Local, and Karachaev breeds displayed 3.82–6.52% of ROH segments with lengths >16 Mb. Considering the pastoral system, such a genetic pattern is most likely the result of demographic decline rather than recent inbreeding and selection pressure.

### Russian Local Goat Breeds: National Variation, Developmental History, and Genetic Connections With Goat Populations From AdaptMap

The postdomestication routes from Eastern Anatolia and Southern Zagros to Africa, Europe, and further in the New World were reconstructed using mitogenomes (Pidancier et al., 2006; Colli et al., 2015), microsatellites (Cañón et al., 2006), and SNP markers (Colli et al., 2018a). Although fossils of goats have been found on the shores of the Caspian Sea and dated to approximately 9000 YBP (Zeder, 2008; Pereira and Amorim, 2010), our results do not elucidate whether the expansion of goats into the territory of modern Russia had occurred in this historic period. Nonetheless, our findings provide a better understanding of the genetic connections of local goats with breeds reared around the world.

Thus, SNP data (Figures 5, 6) demonstrated close genetic relations between Russian local and Turkish breeds. The Angora breed originating in Turkey was imported into many countries and became a transboundary or worldwide breed (Visser et al., 2016; Colli et al., 2018a). The involvement of Angora goats in the creation of some Russian breeds, such as Altai Mountain and Soviet Mohair, with its derivative Dagestan Downy, has been well documented (Orekhov, 1989; Dunin and Dankvert, 2013).

However, earlier admixture events or even the shared ancestry of Russian and Turkish breeds may be hypothesized based on the results of the clustering obtained at K from 2 to 23. Specifically, the detection of the Russian-specific genomic components (gray) in the Kil and Kilis breeds at *K* = 9 and the similarity of the genomic composition with wild bezoar (*Capra aegagrus*) (Figure 6), which was also reported in Turkish goats (Colli et al., 2018a), probably support this assumption. In addition, the clustering with Admixture shows traces in Russian goats of the genomic composition that is present in the Cashmere breed at *K* = 3 (Figure 6) and West Asian breeds at *K* = 7 (Figure 6). Flocks of sheep and goats accompanied human migrations initially for territorial expansion and further for intensive east-west trading (Pereira et al., 2009; Zhao et al., 2017), which probably led to gene flows between breeds reared in different parts of the continent. This theory is supported by the evidence of past admixture between Russian local sheep breeds from the Caucasian Mountains and breeds from Tibet and Iran (Deniskova et al., 2018).

Russian local goats include both those reared on breeding farms with established pedigrees and pastoral goat groups that were raised without data on their developmental history. Data collection for this study included goat populations originating in diverse environmental conditions in remote geographical locations. Thus, the geographical distances varied from 690 km between the Republic of Dagestan and Karachay-Cherkessia Republic to 5500 km between the Republic of Dagestan and Republic of Tyva.

Goats are bred to produce down/cashmere, mohair/fiber, milk, and meat (Novopashina et al., 2019). However, there is no local meat breed in Russia, and the dairy industry is represented mostly by the Saanen breed (Novopashina et al., 2019). Therefore, local breeds have been selected for production traits such as down/cashmere (Orenburg, Altai Mountain, Altai White Downy, and Pridon) and mohair/fiber (Soviet Mohair and Dagestan Downy). This pattern is associated primarily with specific climate conditions in Russia. Thus, the main regions with traditional downy goat breeding are those characterized by a sharply continental climate, such as the Orenburg region and the Altai Republic, where the best local down breeds (Orenburg and Altai Mountain) were developed (Chikalev et al., 2009).

Our results suggest close genetic connections between the Orenburg and Altai Mountain breeds (Figures 3,5 and Supplementary Figure 3). Clustering with Admixture software (Figure 4, at *K* = 4; Figure 6, *K* = 17) also indicates that these breeds have similar genomic backgrounds. Their genetic links might be understood through considering their history.

Orenburg goats were developed by long-term local selection and are famous worldwide for the exquisite quality of their down (Orekhov, 1989). Orenburg craftswomen have traditionally produced the down yarn to knit scarves and shawls by hand (Petrov, 2014). In this regard, the initial selection of the Orenburg breed was not focused on the down length, which is approximately 5.0–5.5 cm (Petrov, 2016). However, recently implemented machine technology requires a 6–7 cm length to produce down yarn, which endangers the Orenburg goats under contemporary circumstances (Petrov, 2016).

The Altai Republic is the homeland for the Altai Mountain breed, which was created by crossing local goats with sires from the Pridon and Angora breeds from 1982 to 1994 (Orekhov, 1989). Although down fibers from the Altai Mountain breed are not as soft and elastic as those produced by the Orenburg breed (Dunin and Dankvert, 2013), the quality of the down is high, and it has good technological qualities (Alkov, 2007; Chikalev et al., 2009). In addition, the Altai Mountain breed easily acclimatizes to new breeding conditions and is widely used to improve aboriginal goat populations in Russian regions and in foreign countries (Mongolia, Kazakhstan) (Alkov, 2007).

We assume a few possible explanations of the genetic closeness between the Orenburg and Altai Mountain breeds that were highlighted in our study. First, the down qualities of the Orenburg goats respond negatively to attempts at improvement by other breeds and are maintained only with pure breeding (Pushkaryov, 2017). However, several reports indicate that Pridon males probably were occasionally used as breed to improve Orenburg goats (Novopashina et al., 2019; Petrov, 2019). Second, both breeds are native to areas situated at the junction of Europe and Asia. Thus, they might have originated from the same local ancestral type of goats, which might have been brought in by nomads or traders. The Orenburg goats have a specific trait: they are successfully reared only in their home region, with an extremely harsh and windy continental climate, and moving into other environmental and geographic conditions results in the almost complete loss of their down characteristics (Pushkaryov, 2017). In this regard, specific signatures of natural selection might result in splitting ancestral types into the Orenburg and Altai Mountain breeds, which correspond to the pattern of breed separation (Figure 4, *K* = 5; Figure 6, *K* = 34).

All analyses and estimates of *F*_*ST*_ show a strong genetic connection between the Altai Mountain and Altai White Downy breeds. The color of the down of the Altai Mountain breed is gray or dark gray. Therefore, a new downy breed was created to produce white down with a length meeting the requirements of combed spinning technology. The Altai White Downy breed was developed on the basis of Altai Mountain females being crossed with Soviet Mohair and Pridon males (Novopashina et al., 2019). However, the Altai White Downy breed demonstrates a strong similarity with its maternal form and has an insignificant share of the Soviet Mohair genetic background (Figure 4, *K* = 5; Figure 6, *K* = 34).

The splitting within the Saanen group genotyped in this study and their differentiation from the same breed from the AdaptMap Project have been revealed by Admixture clustering (Figure 4, *K* = 6; Figure 6, *K* = 23). The Saanen goats for our work were collected in two breeding farms where goats of this breed have been raised for more than 20 years. The first farm originally imported pedigree animals from Holland, and the second farm bought pedigree goats from New Zealand. According to the oral communication of the farmers, both breeding farms currently use only their own sires. In this aspect, the differences accounted for by national selection strategies might have resulted in the pattern of differentiation within the Saanen breed.

Pastoralism is the predominant management system for small ruminants in the Caucasian Mountains. Details of the origin of local goats are scarce. In fact, it is known only that the breeds were created by folk selection (Musalaev et al., 2016) and were extremely diverse and differentiated by horn morphotypes, ear size, and head profiles (Lebel and Zelensky, 1936). SNP data provided an interesting pattern of differentiation of the Karachaev population from other Russian local goats. Such differentiation is indirectly confirmed by the Karachaev breed’s coat colorations (red-brown, yellow reddish, and variegated), which are non-typical of other Caucasian goats. The area of the Karachaev goats overlaps with the habitat of the West Caucasian wild tur (*Capra caucasica*).

*Ex situ* obtaining of viable interspecific hybrids (Lopyrin et al., 1960; Aybazov and Mamontova, 2014) as well as historical remains, providing evidence of an ancient introgression event from a West Caucasian tur-like species (Zheng et al., 2020) might indirectly indicate possibility of introgression in the hybrid zone under natural conditions; however, our results provide no evidence for this additional source of variation in the genome of Karachaev goats. In addition, Karachaev goats showed minimal traces of admixture with the other breeds, which may point to a separate genomic background from the other breeds.

Although Dagestan aboriginal goat populations were assigned to “unimproved” (Dagestan Local) or “improved” (Dagestan Downy), our results suggest admixed origin of both studied Dagestan breeds (Figures 4, 6). The genomic elements of Soviet Mohair are found in the Dagestan Local and are predominant in the Dagestan Downy breed (*K* = 5–6, Figure 4; *K* = 31–34, Figure 6). The Mohair industry has been of great economic importance in the USSR. The import of the Angora breed and improvement of aboriginal coarse wool goats resulted in the development of several breeds that produced semicoarse wool of the Angora type and were well adapted to local environments (Orekhov, 1989; Novopashina et al., 2019). The Soviet Mohair breed created from 1947 to 1962 was considered the best among the new breeds. Dagestan Local goats have been traditionally raised for various products (cheap meat, milk, and goatskins) except for wool and down, which have no practical use because of low quality (Musalaev et al., 2015). Therefore, improvement of local goats with the Soviet Mohair breed began in 1967, and the Dagestan Downy breed was officially established in 1993 (Dunin and Dankvert, 2013). In addition, traces of admixture with the Saanen breed are evident in the Dagestan Local breed (*K* = 3–6 Figure 4; *K* = 3–34 Figure 6). The Saanen breed is preferred as an improver for local goat flocks (Musalaev et al., 2016; Musalaev and Palaganova, 2019). Although there are no official recordings on this account, the unchecked use of Saanen goats to increase the milk productivity of local goats in Dagestan is plausible.

## Conclusion

Here, we present the first comprehensive study of the genome-wide diversity and population structure of goats originating in Russia. Obvious decreases in effective population sizes were displayed in all local breeds that corresponded to the global tendency in livestock species. The estimated levels of genetic diversity indicators in Russian goats do not indicate a critical situation and are comparable with those obtained in a wide range of goat populations within the AdaptMap project. Considering the AdaptMap data, Russian local breeds demonstrated shared ancestry with breeds of Turkish origin. This pattern probably resulted from past admixture events, which took place within postdomestication routes. The revealed genomic composition and pattern of genetic relationships of selected local breeds were supported by the documented data on their origin. Therefore, local breeds selected for down and mohair traits such as Orenburg, Soviet Mohair, and Altai Mountain form their own clusters and represent separate breeds. A significant share of the Soviet Mohair genetic background is found in both Dagestan breeds. In addition, SNP data provide evidence of the influence of the Saanen breed on genetic structure of the Dagestan Local breed. Our findings show a pattern of genetic differentiation between the Karachaev and other local breeds. To clarify the reasons for such differentiation, studies of Karachaev goats will be continued on a larger sample. Thus, our data contribute to a better understanding of the genetic relationships of goats originating in West Asia and Eurasia. Our results will be useful in developing breeding programs for Russian goat breeds to ensure their effective preservation and sustainable utilization.

## Data Availability Statement

The datasets presented in this study can be found in online repositories. The names of the repository/repositories and accession number(s) can be found below: https://figshare.com/articles/dataset/SNP-based_genotyping_provides_insight_into_the_West_Asian_origin_of_Russian_local_goats/14706429.

## Ethics Statement

The animal study was reviewed and approved by the Commission on the Ethics of Animal Experiments of the L.K. Ernst Federal Research Center for Animal Husbandry.

## Author Contributions

TD, AD, NZ, and GB developed the concept and designed the study. MS and A-MA collected goat samples and provided pictures of some goat breeds. MF and HR conducted the molecular genetic work. AD processed and visualized the SNP data. TD, AD, and NZ analyzed the data. TD, NZ, AD, MS, JS, HR, KW, and GB participated in discussions of the data. TD wrote the manuscript. All authors read and approved the final manuscript.

## Conflict of Interest

The authors declare that the research was conducted in the absence of any commercial or financial relationships that could be construed as a potential conflict of interest.
