# Supplementary material for: SNP-Based Genotyping Provides Insight Into the West Asian Origin of Russian Local Goats
Source: Front Genet. 2021 Jul 1;12:708740. doi: 10.3389/fgene.2021.708740 (PMC8282346; doi:10.3389/fgene.2021.708740)
Supplement: Supplementary file 3 [file Data_Sheet_1.PDF]

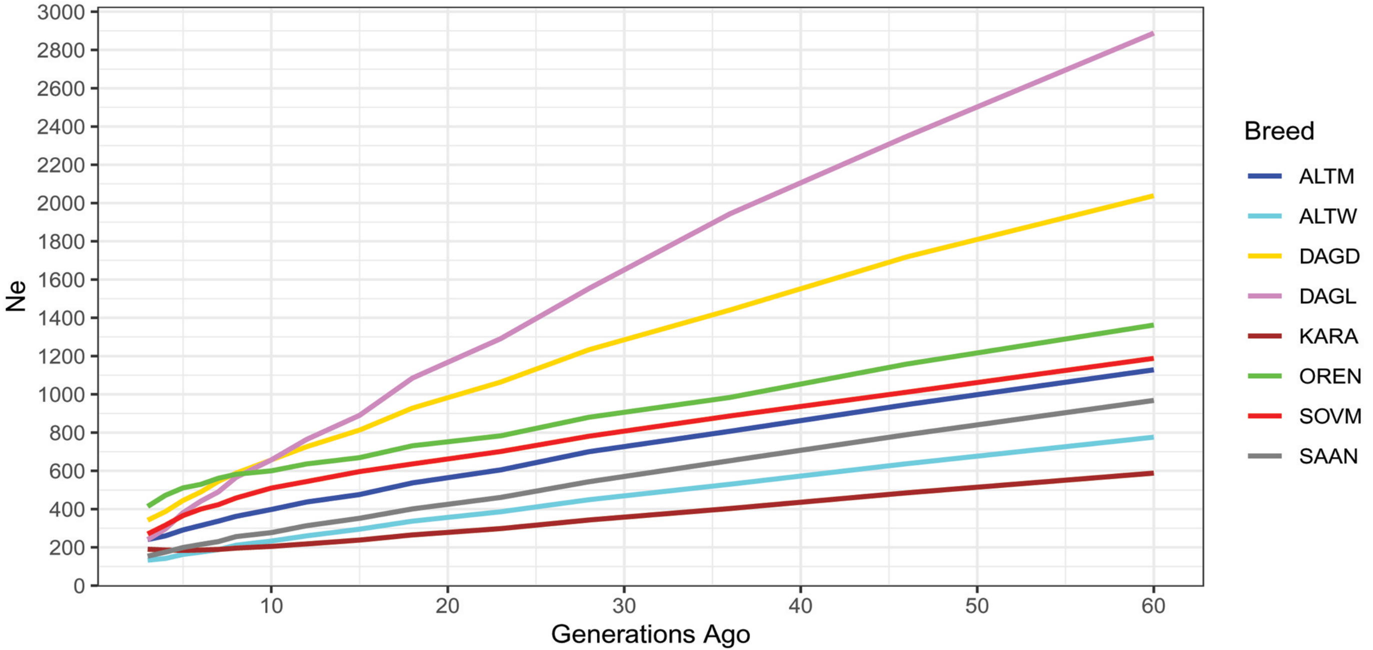


**Supplementary Figure 1.** Effective population size (*Ne*) based on LD estimates for 60 generations ago in Russian goat populations. For a description of the goat breeds, see Table 1.
