# Supplementary material for: SNP-Based Genotyping Provides Insight Into the West Asian Origin of Russian Local Goats
Source: Front Genet. 2021 Jul 1;12:708740. doi: 10.3389/fgene.2021.708740 (PMC8282346; doi:10.3389/fgene.2021.708740)
Supplement: Supplementary file 6 [file Data_Sheet_4.PDF]

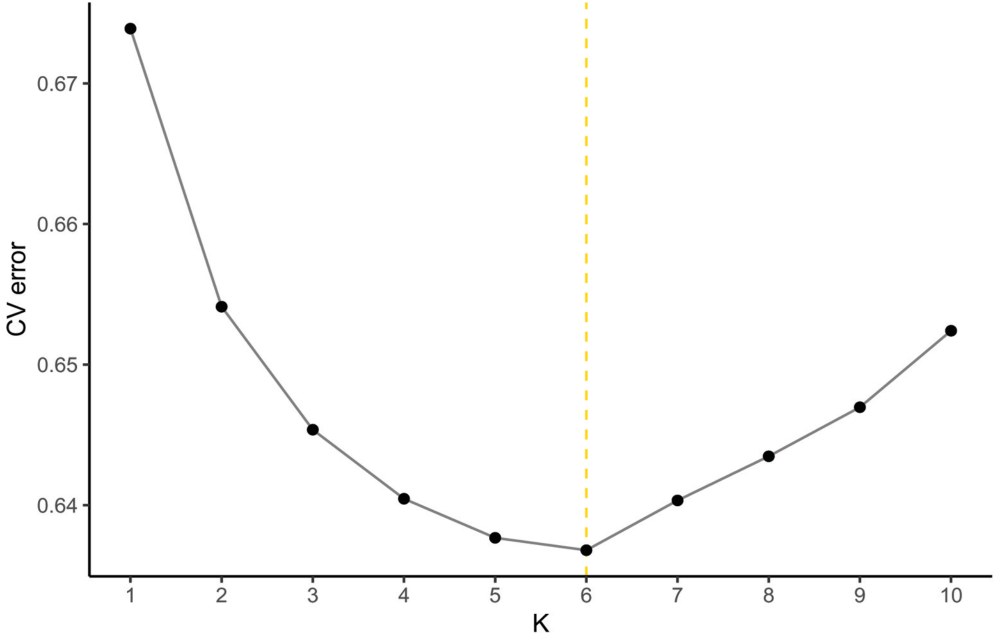

Supplementary Figure 4. Plot of CV-errors of K-values for each K from 1 to 10 for Russian goat populations. K=6 was suggested as the most likely number of clusters.
