# Supplementary material for: SNP-Based Genotyping Provides Insight Into the West Asian Origin of Russian Local Goats
Source: Front Genet. 2021 Jul 1;12:708740. doi: 10.3389/fgene.2021.708740 (PMC8282346; doi:10.3389/fgene.2021.708740)
Supplement: Supplementary file 7 [file Data_Sheet_5.PDF]

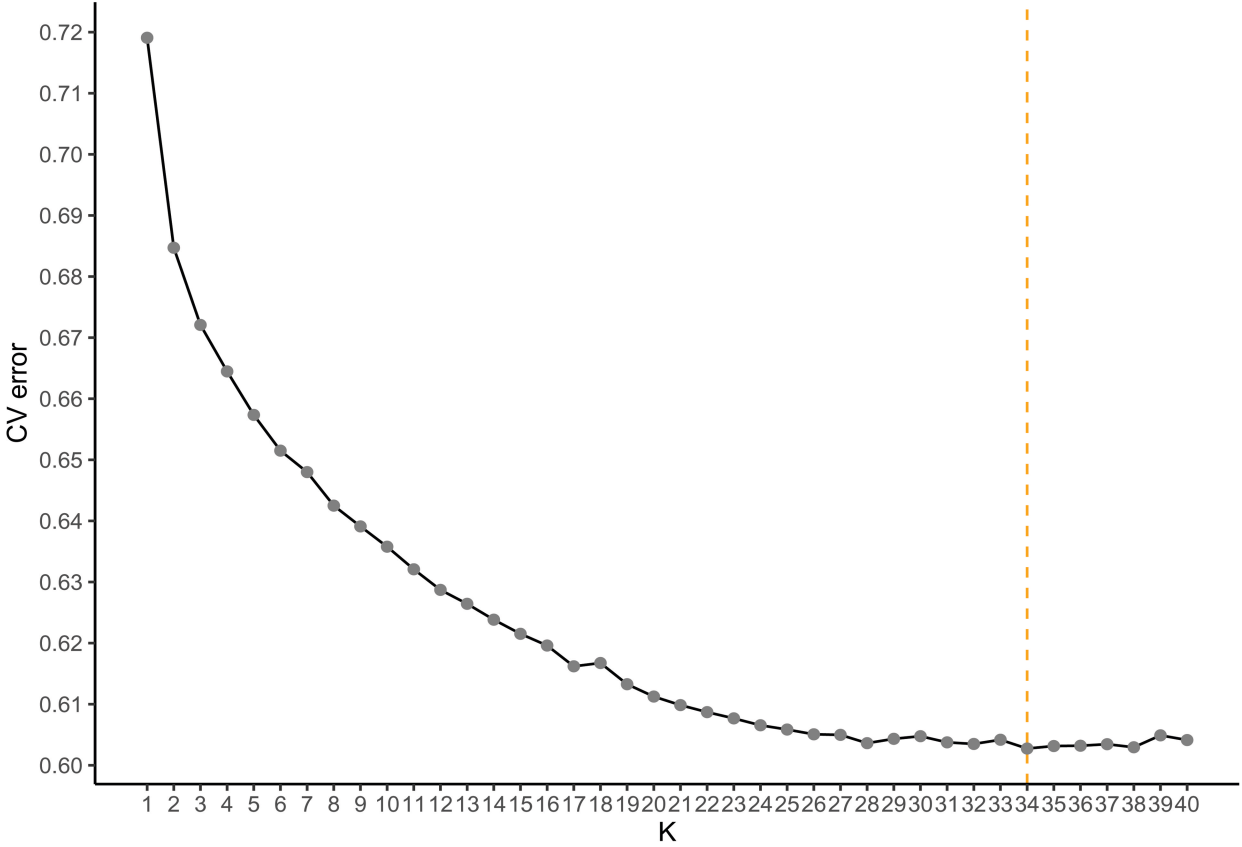

Supplementary Figure 5. Plot of CV errors of K-values for each K from 1 to 40 for Russian and worldwide goat populations from the AdaptMap dataset. K=34 was suggested as the most likely number of clusters.
