# Supplementary material for: SNP-Based Genotyping Provides Insight Into the West Asian Origin of Russian Local Goats
Source: Front Genet. 2021 Jul 1;12:708740. doi: 10.3389/fgene.2021.708740 (PMC8282346; doi:10.3389/fgene.2021.708740)
Supplement: Supplementary file 8 [file Data_Sheet_6.PDF]

Supplementary Table 1. **Population census for goat breeds reared in Russia**

Goat group	Population census				
	Years				
	2000	2005	2010	2015	2019
Alpine	-	-	-	900	3,970
**Altai Mountain**	15,700	11,300	27,300	22,200	10,800
**Altai White Downy**	-	-	-	-	8,300**
**Dagestan Downy**	5,700	16,600	19,500	no data	5,000*
**Dagestan Local**	5,800	16,700	19,600	no data	110,000*
Don (Pridon)	2,000	1,600	-	-	-
**Karachaev**	no data	no data	no data	no data	8,000*
Nubian	-	-	-	-	430
**Orenburg**	16,900	22,800	20,500	17,200	6,500
**Saanen**	-	1,100	6,900	19,900	29,770
**Soviet Mohair**	31,700	88,700	83,300	89,900	28,600
Non-descript	2,800	28,500	8,800	64,800	30,500

* No official recordings are available. The information is based on personal oral communication with goat breeders.

** Altai White Downy was officially recognized in 2016.

References:

Grigoryan, L. N., Hatataev, S. A., and Sverchkova, S. V. (2006). "Sostoyanie kozovodstva Rossijskoj Federacii i ego plemennoj bazy," in Ezhegodnik po Plemennoj Rabote v Ovcevodstve i Kozovodstve v Hozyajstvah Rossijskoj Federacii (2005 god), ed. V.G. Desyatov (Moskva: FGBNU Vserossiiskii nauchno-issledovatelskii institut plemennogo dela Lesnye Poliany), 312–313 (In Russian).

Dunin, I. M., Amerhanov, H. A., Safina, G. F., Grigoryan, L. N., Hatataev, S. A., Hmelevskaya, G. N., et al. (2019). "Kozovodstvo Rossii i ego plemennye resursy," in Ezhegodnik po Plemennoj Rabote v Ovcevodstve i Kozovodstve v Hozyajstvah Rossijskoj Federacii (2019 god) (Moskva: FGBNU Vserossiiskii nauchno-issledovatelskii institut plemennogo dela Lesnye Poliany), 323-325 (In Russian).

Novopashina, S. I., Sannikov, M. Yu., Khatataev, S. A., Kuzmina, T. N., Khmelevskaya, G. N., Stepanova, N. G., et al. (2019) Status and perspective areas for improving the genetic potential of small cattle: scientific and analytic overview. Moskva: Rosinformagrotekh (in Russian).
